# A healthcare-associated outbreak of hepatitis C virus infections attributable to tampering injectable anaesthetic opioids, South Germany, 2017–2018

**DOI:** 10.3389/fpubh.2025.1589562

**Published:** 2025-09-15

**Authors:** Katarzyna Schmidt, Stefanie Böhm, Raffaella Hesse, C.-Thomas Bock, Sebastian Haller, Katharina Katz, Stefan Ross, Jörg Timm, Ruth Zimmermann, Sandra Niendorf

**Affiliations:** ^1^Mycotic and Parasitic Agents and Mycobacteria, Robert Koch Institute, Berlin, Germany; ^2^ECDC Fellowship Programme, Public Health Microbiology Path (EUPHEM), European Centre for Disease Prevention and Control (ECDC), Solna, Sweden; ^3^Infectious Disease Epidemiology, Surveillance and Modelling Unit, Bavarian Health and Food Safety Authority (LGL), Munich, Germany; ^4^Health Department, District Office Donau-Ries, Donauwörth, Germany; ^5^Gastroenteritis and Hepatitis Pathogens and Enteroviruses, Robert Koch Institute, Berlin, Germany; ^6^Healthcare-Associated Infections, Surveillance of Antibiotic Resistance and Consumption, Robert Koch Institute, Berlin, Germany; ^7^Institute of Virology, Essen University Hospital, University of Duisburg-Essen, Essen, Germany; ^8^Institute of Virology, Medical Faculty and University Hospital Düsseldorf, Heinrich-Heine-University, Düsseldorf, Germany; ^9^Department of Infectious Disease Epidemiology, HIV/AIDS, STI and Blood-Borne Infection Unit, Robert Koch Institute, Berlin, Germany

**Keywords:** healthcare-associated outbreak, hepatitis C infection, anaesthetic procedures, phylogenetic analysis, molecular analysis

## Abstract

**Introduction:**

In October 2018, an outbreak of hepatitis C virus (HCV) in southern Germany was communicated to the Robert Koch Institute (RKI). Healthcare-associated transmission during invasive procedures involving a specific anaesthetist at a Bavarian hospital was suspected. The aim was to conduct a retrospective molecular outbreak investigation in order to elucidate the course of the outbreak.

**Methods:**

An exposed patient was defined as a person who underwent a surgical procedure involving the anaesthetist in the Bavarian hospital from May 2016 to April 2018. A probable case was defined as an exposed patient with a positive HCV antibody test result and unknown HCV genotype. A confirmed case represented a probable case with hepatitis C genotype 3 (3a) infection. Descriptive epidemiological and phylogenetic analyses (using four HCV regions: Core, HVR1, NS5A and NS5B) were conducted.

**Results:**

Of the 1,714 exposed patients, to whom HCV testing was recommended, 1,558 (90.9%) responded and were tested, 63 met the definition of a probable case, and 51 of those were confirmed cases. Sequencing data were available for 39 of the 51 confirmed cases. A sample from the anaesthetist was unavailable for further analysis. Phylogenetic analysis revealed close genetic relatedness of all 39 confirmed cases with identified HCV genotype 3a. Phylogenetic results indicated a common source of infection.

**Discussion:**

To prevent healthcare-associated HCV transmission during anaesthetic procedures, protocols must document the amount of medication used and discarded. Regular staff testing and storing of clinical samples are also crucial for timely outbreak analysis and response.

## 1 Introduction

Hepatitis C is a liver inflammation caused by hepatitis C virus (HCV) predominantly spreading through the parenteral route. An acute infection leads to a chronic course in 50–85% of individuals, potentially progressing to liver cirrhosis and hepatocellular carcinoma (1). Treatment options have improved since the introduction of direct acting antivirals with cure rates of 90% and more (2, 3).

The HCV genome is an enveloped, positive-strand RNA of about 9.6 kb with a single open reading frame encoding 3,000 amino acids and cleaved into structural (core, E1, E2) and non-structural (p7, NS2, NS3, NS4A, NS4B, NS5A, NS5B) proteins (4). Seven HCV genotypes and numerous subtypes have already been described (5), but only a few of them are currently circulating (1a, 1b, 2a, 3a, 4) in high income countries (5–7).

Healthcare-associated HCV transmission usually involves contaminated equipment (8–10) or unsafe injection practices (11, 12). Transmission can also occur via healthcare providers diverting drugs through unsafe injections (13–15). Typically, drug diversion involves a healthcare worker (HCW) misusing narcotic drugs intended for patients, also called “tampering” (16). HCV transmission could occur if the HCW is infected with HCV.

In June 2018, a local public health authority (LPHA) in Bavaria received a report of hepatitis C infection in an anaesthetist, whose employment at a local hospital had ended in April 2018 due to termination agreement. In October 2018, the LPHA was notified about a cluster of three new HCV infections of unknown origin in patients without clear risk behaviours as reported by a local general practitioner (GP). The LPHA investigation revealed that the affected patients had surgeries at the local hospital where the anaethetist was previously employed, and that these operations involved the anaesthetist in question. Therefore, the suspected transmission of HCV was a link between the patients and the anaesthetist. When an epidemiological link to the anaesthetist emerged, the LPHA, together with the local hospital, launched an extensive case finding and testing campaign to find further infected patients and determine the extent of the outbreak.

Phylogenetic analysis of viral sequences is often used to determine which samples are part of an outbreak and – when samples from suspected index persons are available – to identify the source of healthcare-associated HCV outbreaks (17–20). Determining the degree of genetic relatedness between viral isolates from the probable source of infection and from known cases provides valuable evidence to support and/or rule out hypotheses about possible transmission pathways. Here, we applied this phylogenetic method combined with epidemiological contact tracing data to demonstrate its utility in a retrospective analysis of a large hospital-acquired HCV outbreak associated with anaesthetic procedures in Germany.

## 2 Methods

### 2.1 Case finding

The case-finding period was delineated based on the anaesthetist’s previous HCV tests and the dates of anaesthetist’s employment at the local hospital. The anaesthetist’s last negative HCV test result during occupational health examinations, was in November 2016. Anti-HCV seroconversion occurs on average eight to 11 weeks after infection (21), but may take up to six months (22). Therefore, the start of the case-finding period was set as May 2016, which was six months before the last negative test. The end of the case-finding period was set as the April 24th 2018, which was the anaesthetist’s last working day at the local hospital. An acute HCV genotype 3 infection in the anaesthetist was confirmed in June 2018, with antiviral therapy initiated one month later. A negative PCR result was recorded in October 2018. Unfortunately, none of the anaethetist’s samples were available for this investigation, as they were discarded in the laboratory and not retained for further analysis.

Hospital staff reviewed over 10,000 surgical protocols from the defined period to determinate the involvement of the anaesthetist in question. Certain procedures, including major surgeries, were linked to potential exposure, while others such as central lines insertions, were excluded based on interviews with anaesthetists, clarifying the scope of procedures considered. A total of 1,714 patients, whose surgical procedures involved the anaesthetist during the case-finding period, were contacted via letter by the hospital and LPHA to inform them about the outbreak and to invite them for hepatitis C screening. Testing for other bloodborne pathogens including hepatitis A, hepatitis B and HIV was not offered, as these infections had been ruled out based on prior examinations of the anaesthetist, which yielded negative results. Among patients contacted anti-HCV status was determined. None of the individuals had a previously known hepatitis C infection. Additionally, all employees (doctors, nurses as well as operating theatre and cleaning staff) working in the hospital’s operating theatre were tested for an HCV infection, with the exception of 6–8 former employees who could not be located or who refused to provide consent. No additional hepatitis C-positive staff member was identified.

### 2.2 Case definitions

An exposed patient was defined as a person who underwent a surgical procedure involving the anaesthetist in the local Bavarian hospital from May 2016 to April 24th 2018. A probable case was defined as an exposed patient with a positive HCV antibody test result and unknown HCV genotype. A confirmed case was defined as a probable case where infection with hepatitis C genotype 3 (3a) was confirmed via genotyping. Phylogenetic analyses were conducted on confirmed cases with available serum samples.

### 2.3 Descriptive analysis

**Figure 1:**
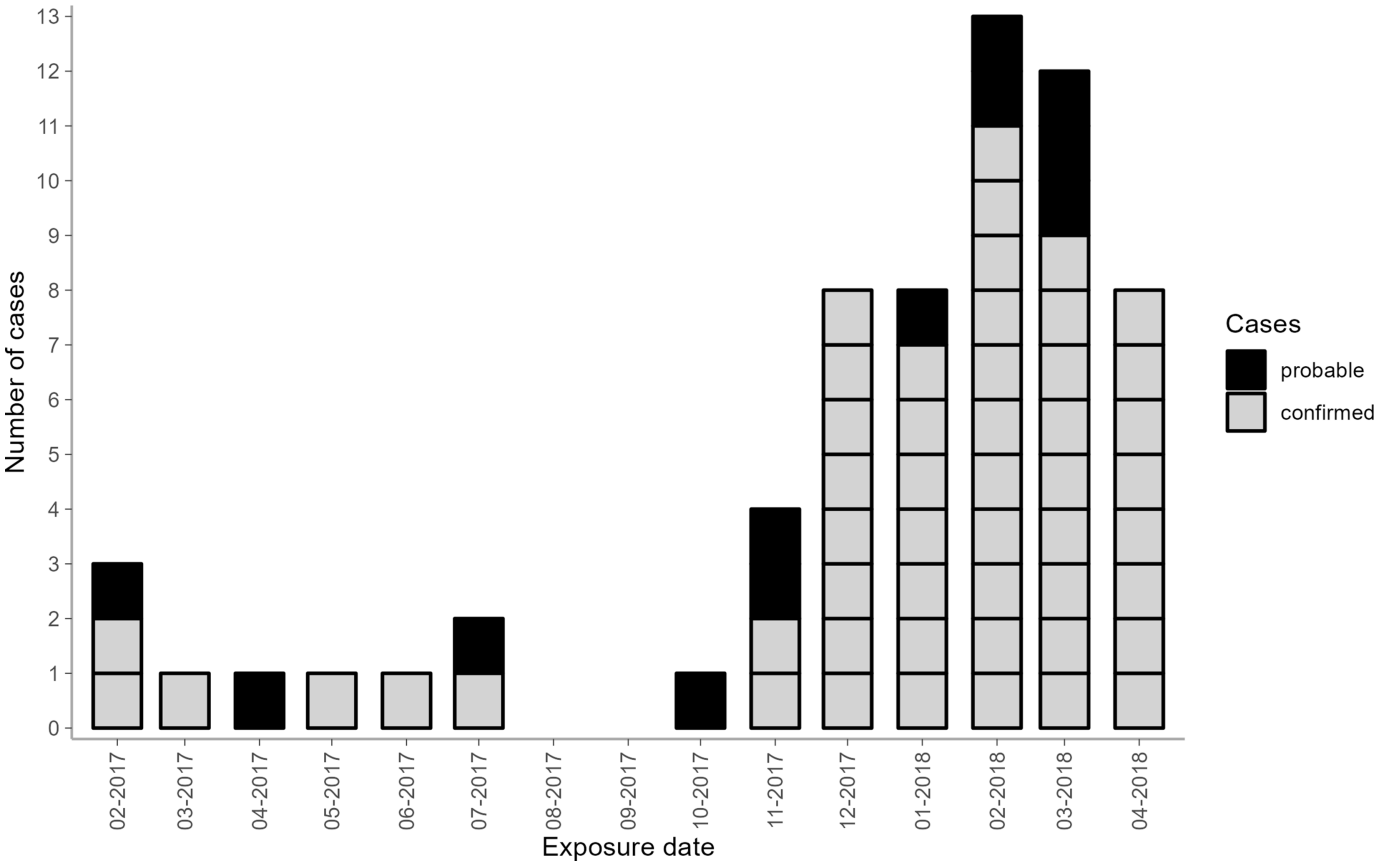
Number of probable and confirmed cases by month of exposure belonging to the hospital-acquired HCV outbreak, Germany 2017–2018. Black—probable cases; grey—confirmed cases. Two confirmed cases had more than one date of surgery recorded (Case 1: 2017–02 and 2018–03; Case 2: 2017–12 and two dates in 2018–01). In these cases, the first date of exposure was used.

Detailed demographic and clinical information were collected in a linelist. An epicurve was constructed using the exposure date (date of surgery) (Figure 1). Descriptive epidemiological analysis was performed for basic demographics (gender and age) using R (version 4.1.3). Due to the retrospective nature of the analysis, age and age groups were calculated based on the time of HCV testing.

### 2.4 HCV testing

Exposed patients had HCV antibody tests performed at the local hospital or at a private laboratory initiated by the patients’ GPs. Positive HCV antibody samples from all probable cases were sent to the Hepatitis C National Reference Laboratory (NRZ) University of Duisburg-Essen for HCV viremia testing. The Abbott real time HCV assay (Abbott, Des Plaines, United States) was used for quantification of HCV-RNA and genotyping following the manufacturer’s procedure.

Serum samples of 44 out of the 51 confirmed cases were available and were sent to the RKI laboratory for further investigation. The RNA was extracted from 140 μL of the serum using the Qiagen viral RNA extraction kit (Qiagen Hilden, German) according to the manufacturer’s instructions. Reverse transcription was performed using SuperScript^™^ IV Reverse Transcriptase (200 U/μL) (Thermo Fisher, Waltham, United States) at the following conditions: denaturation at 65 °C for 5 min., annealing at 23 °C for 10 min., cDNA synthesis at 50 °C for 6 min., 55 °C for 6 min., 60 °C for 6 min., and termination at 80 °C for 10 min. Hepatitis C genotype confirmation was performed using SuperScript III Platinum One-Step qRT-PCR Kit (Thermo Fisher) with primers and TaqMan probe specific for HCV 3 genotype (Table 1). PCR conditions were as followed: 50 °C for 5 min., 55 °C for 10 min., 95 °C for 1 min., and then 45 cycles of PCR amplification at 95 °C for 15 s., and at 58 °C for 45 s. Prior to the Sanger sequencing, four independent nested PCR tests were performed using primers specific for four HCV genotype 3a regions (core, hypervariable-HVR1, nonstructural 5A-NS5A and nonstructural 5B-NS5B) described in Table 1. PCR products were visualized on a 1x TAE 1.5% agarose gel using 100 base pairs (bp) DNA Ladder (Thermo Fisher). PCR products (core: 230 bp, HVR1: 523 bp, NS5A: 679 bp and NS5B: 674 bp) were cleaned by ExoSap-IT (Thermo Fisher) and sequenced with corresponding PCR primers using BigDye Terminator v3.1 Cycle Sequencing Kit (Thermo Fisher) by the Method development, research infrastructure and information technology department at the RKI.

**Table 1:** Primers and probes used for amplification and sequencing in the hospital-acquired HCV outbreak, Germany 2017–2018.

Region	Name	Sequence (5′–3′)	Position (nt)
5’NCR	HCV-255 (s)	AGYGTTGGGTYGCGAAAG	258–275
	HCV-256 (as)	CACTCGCAAGCRCCCT	296–311
	HCV-TM5	FAM-CCTTGTGGTACTGCCTGA-MGB	277–294
Core	HCV-401 (s)	GTGCCCCGGGAGGTCTGT	309–327
	HCV-402 (s)	GTAGACCGTGCATCATGAGCAC	326–347
	HCV-403 (as)	CGCTCCGACGCGCCTTGG	536–553
HVR1	HCV-407 (s)	ATGGCTTGGGATATGATGATGAA	1,291–1,313
	HCV-408 (as)	CTAGGTGCGTAGTGCCAGCA	1798–1817
NS5A	HCV-292 (s)	GCTGAGTTCTCTAACTGTCACAAG	6,198–6,221
	HCV-293 (s)	ACTGTCACAAGTCTGCTCCGG	6,211–6,231
	HCV-294 (as)	GATGAGCTTGCCTCTGATGGA	6,942–6,962
	HCV-295 (as)	GGTCTCTCAACATCGAGGTCAGC	6,867–6,889
NS5B	HCV-271 (s)	ACCACATCMRSTCCGTGTGG	7,979–7,998
	HCV-272 (s)	TCCGTGTGGRARGACYTSCTRGA	7,990–8,012
	HCV-275 (as)	CTSGTCATAGCYTCCGTGAA	8,644–8,663
	HCV-305 (s)	CTCCGTMTGGGAGGACTTGC	7,989–8,008

### 2.5 Phylogenetic analysis

**Figure 2:**
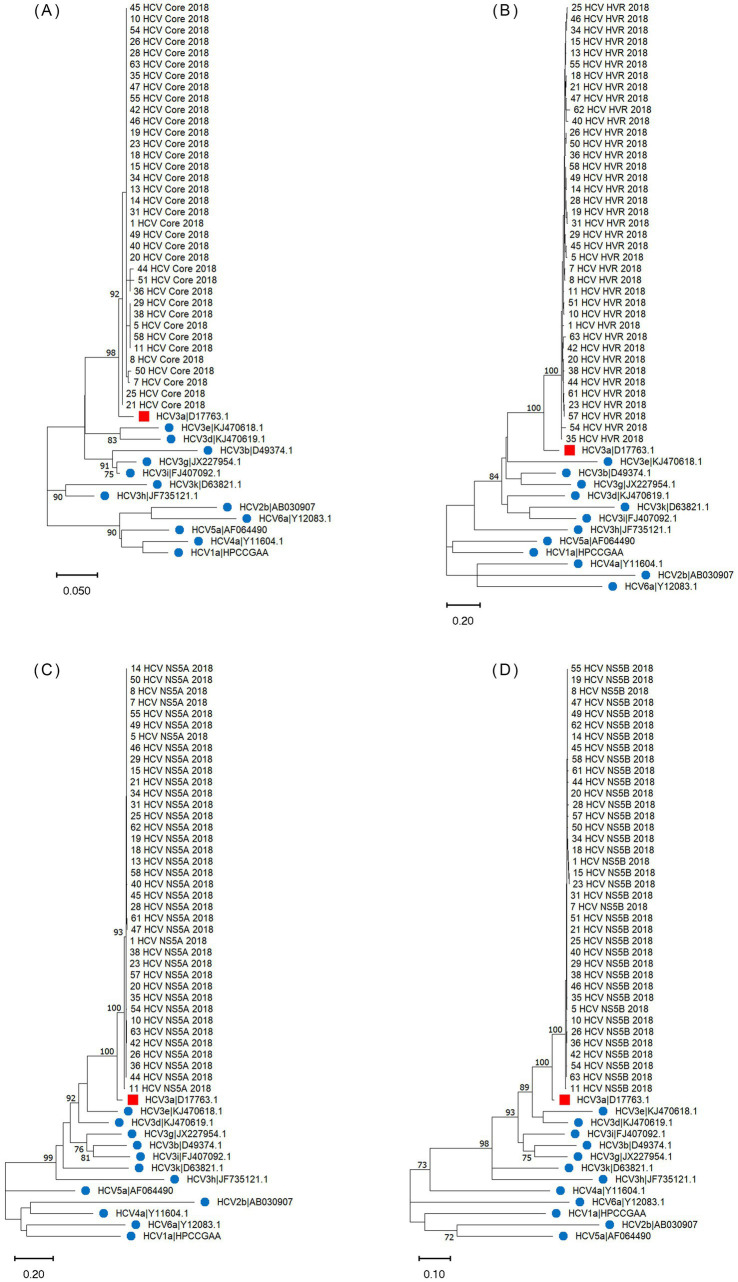
Phylogenetic analysis of HCV isolates based on core **(A)**, HVR1 **(B)**, NS5A **(C)** and NS5B **(D)** regions of the hospital-acquired HCV outbreak, Germany 2017–2018 from 39 confirmed cases. Red square—reference strain for HCV genotype 3a; blue circle—reference strains for others HCV genotypes. Only bootstrap values greater than 70 are highlighted on the trees. GenBank numbers of all reference sequences used in the study: HCV1a (HPCCGAA), HCV2b (AB030907), HCV3a (D17763.1), HCV3b (D49374.1), HCV3d (KJ470619.1), HCV3e (KJ470618.1), HCV3g (JX227954.1), HCV3h (JF735121.1), HCV3i (FJ407092.1), HCV3k (D63821.1), HCV4a (Y11604.1), HCV5a (AF064490), and HCV6a (Y12083.1).

For 39 confirmed cases, isolates were available for sequencing. Phylogenetic analysis was performed on 36 core sequences (225 bp), 39 HVR1 sequences (504 bp), 38 NS5A sequences (657 bp) and 38 NS5B sequences (653 bp). The alignments for each of the regions were performed using MUSCLE 3.8.425 in Geneious software (v2021.2.2) with default parameters (maximum of 8 interactions). Phylogenetic trees were constructed by the maximum likelihood (ML) method with bootstrap 1,000 using the software MEGA11 (v11.0.11) and 13 reference sequences (Figure 2). For each region, the model based on the lowest Bayesian Information Criterion scores was selected. The Kimura 2-parameter model with invariant sites (K2 + I) was used for HCV core region, and the Tamura 3-parameter model with gamma distribution and invariant sites (T92 + G + I) was used for HVR1, NS5A and NS5B regions. The remaining parameters were set as defaults. Bootstrap values higher than 70 were shown at the nodes of the phylogenetic trees.

Given that no serum sample from the anaesthetist was available, a most recent common ancestor (MRCA) sequence for each genomic region was reconstructed using Arning approach (23). This method reconstructs a probable ancestral sequence by aligning viral genomes from confirmed cases and selecting the sequence with the fewest mutations relative to the outbreak samples. The MRCA was then used as a reference to calculate the number of single nucleotide polymorphisms (SNPs) distances in the 39 confirmed cases with available sequencing data. SNP distances were interpreted in terms of genetic relatedness. Generally, a low number of SNPs indicates a higher degree of similarity between the MRCA and a confirmed case suggesting a closer genetic relationship within the outbreak. To assess whether the number of cumulative SNPs increased with time, correlation coefficient and the R squared values for the relationship between the SNPs count and the exposure and the testing dates were calculated.

## 3 Results

### 3.1 Descriptive findings

Of the 1,714 eligible patients contacted, 1,558 were tested for HCV antibody screening (response rate = 90.9%). Among them, 63 probable cases were identified, with 51 cases confirmed by genotyping. The remaining twelve probable cases were HCV-RNA negative and genotyping was unable to be performed. Among the 51 confirmed cases, the epidemiological investigation revealed no alternative sources of hepatitis C exposure besides surgeries involving the anaesthetist in question. Thus, the timeframe from the first to the last exposure dates of probable and confirmed cases ranged from February 2017 until April 2018 (Figure 1). In the two cases with more than one surgery, only the first date of surgery was reported and used in further analysis. Nine cases had exposure dates between February 2017 and July 2017. After October 2017, there was a sharp uptick in the probable and confirmed cases, peaking at 13 cases in February 2018. Twelve cases had exposure dates in March 2018 and eight cases in April 2018. Confirmed cases were equally distributed among males and females (25:26) and were mostly adults in older age groups (51–90 years old) (Table 2).

**Table 2:** Number of confirmed cases by age group and gender involved in the hospital-acquired HCV Germany 2017–2018.

Age groups	Male	Female	Total
11–20	2 (67%)	1 (33%)	3 (5.9%)
21–30	1 (50%)	1 (50%)	2 (3.9%)
31–40	2 (100%)	0	2 (3.9%)
41–50	0	7 (100%)	7 (13.8%)
51–60	7 (70%)	3 (30%)	10 (19.6%)
61–70	6 (50%)	6 (50%)	12 (23.5%)
71–80	4 (44%)	5 (56%)	9 (17.6%)
81–90	3 (50%)	3 (50%)	6 (11.8%)
Total	25 (49%)	26 (%)	51 (100%)

### 3.2 Laboratory findings

**Figure 3:**
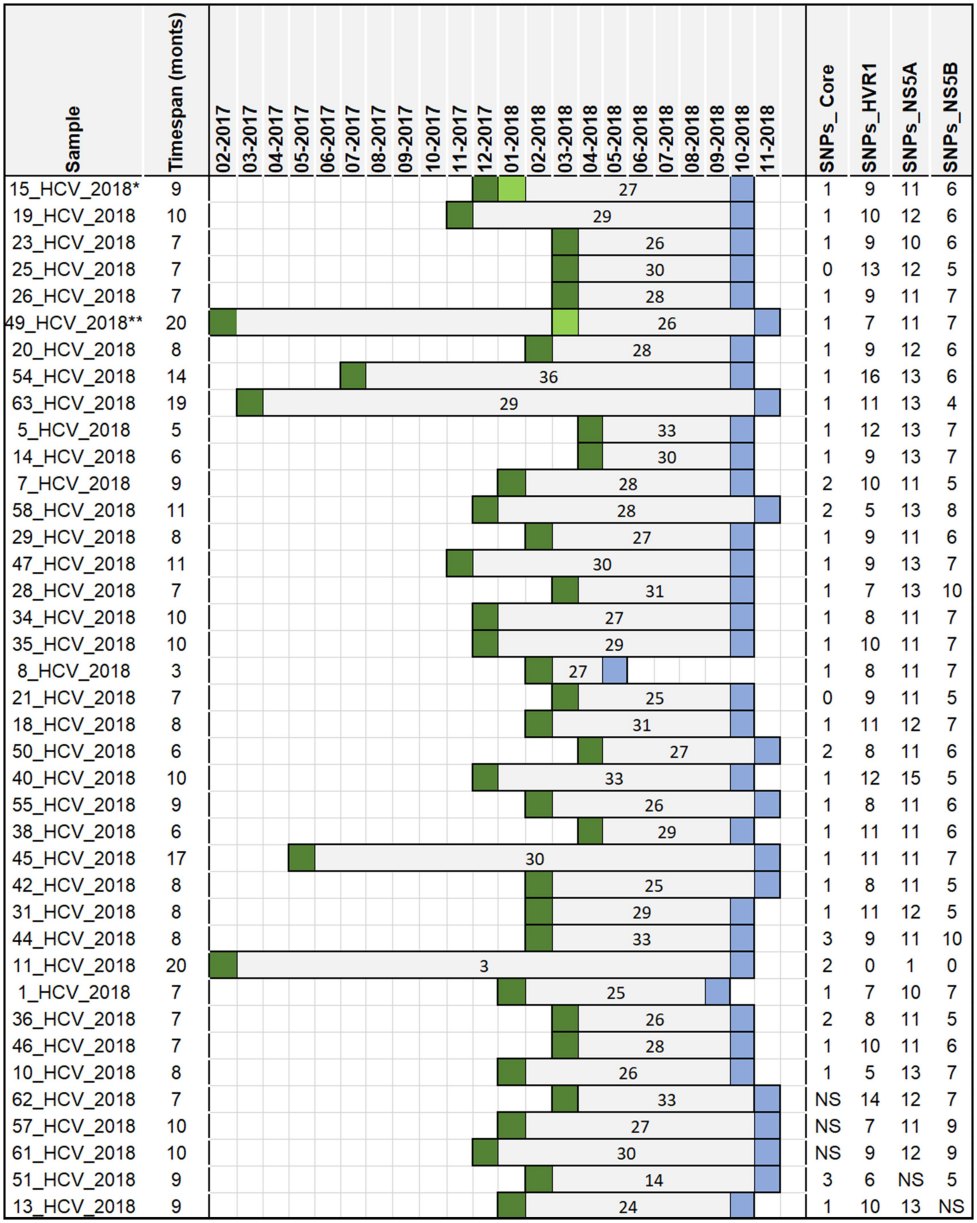
The timespan between the date of exposure and the date of testing in a hospital-acquired HCV outbreak, Germany 2017–2018 for 39 sequenced samples from 39 confirmed cases. The ancestor sequences used to assess number of SNPs include 21_HCV_2018 for core region and 11_HCV_2018 for the remaining regions. The number inside the bars indicates the cumulative number of SNPs for all regions. Dark green square—exposure dates (surgery dates); light green square—other exposure dates in patients with more than one surgery date (Case 1**: 2017–02 and 2018–03; Case 2*: 2017–12 and two dates in 2018–01); blue square—testing dates (initial anti HCV screening); NS-no sequence data available.

Of the 51 confirmed cases, six were identified with genotype 3, and 45 with genotype 3a. Serum samples of 44 of the 51 confirmed cases were available for sequence analysis, with sequence and epidemiological data concordant in 39 cases. Phylogenetic analyses revealed that all 39 confirmed cases were closely related and belonged to the HCV genotype 3a (Figure 2), which is the same genotype previously identified in the anaesthetist (written communication from the local health authority). The examined samples clustered with a high level of confidence, supported by high bootstrap values (greater than 90%) for each region, indicating close genetic relatedness between the samples. In the remaining 12 confirmed cases without sequence data, it was not possible to determine with certainty that they were highly related, as their classification was based solely on hepatitis C genotype 3 (3a) infection and not on phylogenetic analysis. The Arning’s MRCA sequence reconstruction approach revealed that all viral sequences were closely related, thus almost all of them could be used as an ancestor in further analyses. Therefore, the sequences that were the closest to the MRCA (21_HCV_2018 for core region and 11_HCV_2018 for the remaining regions) were used as reference to calculate the number of SNPs distances in the HCV isolates. In total, we identified 1,073 SNPs across four genomic regions (Core: 43, HVR1: 354, NS5A: 435, NS5B: 241) with an average 27 SNPs per sample (range 3–36 SNPs per sample) in the HCV isolates from the 39 confirmed cases (Figure 3). The range of nucleotide genetic distances between the PCR amplicons to the genomic regions were as follows for core: 0–3, HVR1: 0–16, NS5A: 1–15, and NS5B: 0–10. The timeframe from exposure to testing dates spanned from February 2017 to November 2018 (Figure 3), with exposure-to-testing periods ranging from three to 20 months, and averaging nine months. The statistical test showed no correlation between the number of SNPs and the exposure date and testing date (Table 3).

**Table 3:** Regression statistics of the relationship between the number of SNPs and exposure dates and testing dates in the hospital-acquired HCV Germany 2017–2018.

HCV region/regression statistics	Exposure date		Testing date	
	Correlation coefficient	R squared	Correlation coefficient	R squared
Core	−0.054931345	0.003017453	0.131796245	0.01737025
HVR1	0.191759664	0.036771769	0.045887795	0.00210569
NS5A	0.346800714	0.120270735	0.077825277	0.006056774
NS5B	0.332666684	0.110667123	−0.000394761	1.55836E-07

## 4 Discussion

This report describes the largest known hospital-acquired HCV outbreak in Germany, linked to drug diversion by an infected HCW. The findings indicate that at least 51 patients were likely infected by the same anaesthetist during anaesthetic procedures. This investigation retrospectively describes the transmission cluster of HCV-affected patients, and ascertains the genetic relatedness of the HCV isolates from the confirmed cases. Genotyping, together with epidemiological data, identified 51 confirmed cases with HCV genotype 3 (3a) infection from 63 probable cases. In-depth phylogenetic analysis of four HCV regions in a sub-sample of confirmed cases suggested that all were infected by the same single viral strain of HCV genotype 3a, indicating close genetic relatedness, supported by high bootstrap values. While the anaesthetist’s sample was unavailable for analysis, no evidence of other HCV-positive HCWs or epidemiological links between the confirmed cases were found. Based on these findings, we hypothesize that the probable source of infection was an HCV-positive anaesthetist involved in all the confirmed cases; and the suspected transmission route was the contamination of syringes and/or needles through misuse by the infected anaesthetist prior to administration to the patient. However, the study’s limitations prevent us from conclusively verifying these hypotheses.

The outbreak was detected in October 2018, when a Bavarian LPHA was notified of three newly diagnosed cases of hepatitis C in patients with unknown exposure, and an epidemiological link to one local hospital was identified. An outbreak investigation was launched using epidemiological analysis and anti-HCV screening for patients who underwent surgery between May 2016 and April 2018. The earliest surgery date was February 2017. Knowing that spontaneous clearance of acute HCV associated with genotype 3 is common, at least in the first 3 months after infection (24, 25), the delay for case-searching may have led to undiagnosed and/or missed cases. Here, the exposure-to-testing period for probable cases ranged from 1 to 21 months (average 9 months), which means that some patients might have developed a chronic condition before the implementation of treatment. However, no clinical information regarding the HCV infections, symptoms and treatment were available. Due to HCV’s long incubation and often asymptomatic nature, healthcare-associated HCV outbreaks are frequently identified with a significant delay. Consequently, determining the exposure period and transmission route may be challenging or even impossible, potentially resulting in undiagnosed and therefore untreated cases. Despite challenges in distinguishing acute from chronic HCV infection, implementing recommendations for managing HCV-infected HCWs in clinical settings remains essential. For instance, regular mandatory testing of HCWs by occupational health can support early detection of infected HCWs, and by that, reduce risk of transmission during exposure-prone procedures to patients. The German Association for the Control of Viral Diseases (DVV) prohibits high-risk activities for HCWs with HCV viral loads >25,000 IU/mL, and allows those with viral loads <250 IU/mL to perform such activities with precautions (e.g., double gloves) (26). In addition, early antiviral therapy is recommended for infected HCWs and patients (25) to prevent liver damage and transmission.

We hypothesized that a longer exposure-to-testing interval would result in a higher number of cumulative SNPs per sample due to the accumulation of mutations over time. However, data from the 39 confirmed cases despite varying intervals showed no consistent pattern (Figure 3). For example, patient number 8_HCV_2018 (Figure 3), with the shortest period (3 months) between exposure and testing dates, had 27 cumulative SNPs identified similar to the number found in patient 49_HCV_2018 (26 SNPs) despite the longer interval (20 months). In contrast, two patients (11_HCV_2018 and 63_HCV_2018), with similar interval between exposure and testing dates (19 and 20 months) showed substantial differences in SNPs counts (3 versus 29 SNPs respectively). The reconstructed sequence of patient number 11_HCV_2018 demonstrated the closest similarity to the MRCA (exposure date: February 2017), thus serving as the common ancestor for most of the HCV regions. Despite the expectation that cumulative SNPs would increase over time, the linear regression revealed no significant correlation between the total number of SNPs and time (measured by the exposure date and the testing date). However, the lack of the viral sequence from the suspected anaesthetist’s sample prevented a direct comparison with confirmed cases, limiting our ability to reconstruct transmission chains with higher certainty. Furthermore, the minimal genetic variation among available sequences restricted temporal inferences, making it difficult to determine precise transmission timelines.

In western European countries, the distribution of HCV genotypes is relatively uniform. A recent study showed that the predominant HCV genotypes in Germany are genotype 1 (47%) and genotype 3 (46%) (27). HCV genotype 3a is commonly found among people who inject drugs (28–31). Healthcare-associated HCV outbreaks attributable to narcotic diversion or unsafe injection practices have been reported in the United States (14, 32, 33), and in several European countries, including France (11) and Spain (19, 20). These cases reveal common vulnerabilities such as delayed outbreak recognition, insufficient monitoring of anaesthetic drug access, and institutional failure to detect diversion behaviours. Hatia et al. (16) conducted a meta-analysis of 46 studies published between 1990 and 2012 describing nosocomial HCV outbreaks caused by HCWs who diverted injectable opioids. Their findings showed that the HCV transmission risk from drug diversion was substantially higher compared to surgical exposure, underscoring the importance of targeted prevention strategies.

Key aspects to prevent drug diversion outbreak involve proactive measures and protocols in healthcare settings to minimize risk and ensure patient safety (32). Common approaches include monitoring access to dispensing system for unusual patterns related to illegal drug procurement, using machine learning to assess diversion risk (34), pre-screening of healthcare staff with medical record surveillance, and establishing dedicated drug diversion prevention teams. Schaefer and Perz (14) emphasized the importance of involving external resources, such as state health departments and local law enforcement, to prevent and respond to drug diversion outbreaks. Lahey et al. (35) described an outbreak of drug diversion in New Hampshire (United States) linked to cardiac technical who infected dozens of patients through repeated drug diversion across multiple hospitals. This case illustrates the ethical and structural risks associated with failing to report known diversion behaviours. In response, the authors proposed the implementation of a centralized national reporting system to support healthcare institutions in fulfilling their ethical obligation to protect patient from such preventable harm. Similarly, our investigation relied on collaboration with local, regional, and state health authorities, underscoring the importance of transparency, reporting structures, and system-wide learning in responding to and preventing future outbreaks.

The molecular identification of HCV sequence clusters can speed up public health response (18). Two prominent molecular approaches have been proposed to investigate the HCV transmission clusters in outbreaks settings (18, 36). While many uses next-generation sequencing to track transmission chain, we used Sanger sequencing and phylogenetic analysis of four HCV regions, which proved effective for this investigation.

The study had four sample-related limitations. First, the anaesthetist’s sample was unavailable. Thus, we could not include it in the phylogenetic analysis to definitively confirm that the anaesthetist was a source of the outbreak. The lack of this sequence data limits our ability to establish a direct genetic link between the aneasthetist and other cases, which affects the strength of causal conclusions regarding transmission pathways. However, despite this limitation, our study provides strong epidemiological and genomic evidence supporting transmission events. The available viral sequences from confirmed cases show high genetic relatedness, indicating a common transmission source. Although, the missing sample limits a complete phylogenetic reconstruction, the clustering of cases – considering their genetic similarity as well as temporal and spatial proximity through surgeries performed at the same hospital by the anaesthetist in question – supports the common source of transmission. To enhance genomic investigations in future outbreaks, the systematic collection and long-term storage of all relevant clinical samples should be prioritized. Additionally, further analysis (e.g., molecular clock) to determinate the HCV genome’s evolution rate and estimate the exact date of infection, was not possible. This would have helped to narrow the exposure period and reduce the number of patients needing testing. However, the lack of the viral sequence data from the anaethetist prevented the establishment of reliable priors for our analysis. Furthermore, the genetic similarity among the available sequences from confirmed cases resulted in minimal sequence variation, limiting the resolution of temporal inferences needed to accurately estimate infection timing. Second, sequencing data were missing for 12 confirmed cases, all of which had the same exposure period as the other confirmed cases falling within the case-finding period, and underwent surgery at the same hospital involving the suspected anaethetist. Although, the lack of sequence data unable their direct inclusion in the phylogenetic analysis, their strong epidemiological link suggests they would have clustered with the outbreak cases, already supported by a high bootstrap value (greater than 90%). This limitation highlights the need for improved communication and/or cooperation between parties involved, as well as the importance of storing HCV-positive samples for extended period to facilitate future genomic investigations. Third, clinical information, including symptomatology, liver function outcomes, and antiviral treatment, was not available for the confirmed cases. While such data would have strengthened the clinical interpretation of our findings, their absence does not affect the genetic and epidemiological conclusions of the study. Future investigations would benefit from integrating clinical and genomic data to provide a more comprehensive understanding of transmission dynamics and disease outcomes. Lastly, the sampling dates (day of sample collection) were missing for almost 50% of the sequenced HCV isolates. In order to disclose the number of SNPs that evolved within a timespan (exposure-to-sampling date) we used a proxy for the sampling date. This likely introduced little change to our findings, as the testing date (the date when the samples were tested positive) is the closest date to the sample collection date to the best of our knowledge.

This study describes a large hospital-acquired HCV outbreak in Germany likely caused by drug diversion. Molecular analyses indicated that all the confirmed cases were closely genetically related and likely stemmed from the same infection source. Early outbreak detection of chronic infections like HCV is challenging, making preventive measures crucial. This includes maintaining staff restrictions to accessing controlled substances, the monitoring of access to dispensing systems, and establishing drug diversion prevention teams. Furthermore, we recommend regular testing for HCV and other blood-borne infections of all HCWs, in particular of those involved in anaesthetic procedures and opioids handling. Preventing the transmission of infections from infected staff to patients, whether HCV or others-is crucial. Therefore, clear guidelines, monitoring and control systems of drug diversion should be implemented in hospitals. Importantly, positive samples should be stored for further testing to support rapid and effective outbreak investigations, if needed.

## Data Availability

The original contributions presented in the study are included in the article/supplementary material, further inquiries can be directed to the corresponding author/s.
